# Implementation of long-term non-participant reminders for flexible sigmoidoscopy screening

**DOI:** 10.1016/j.pmedr.2020.101308

**Published:** 2021-01-04

**Authors:** R.S. Kerrison, A. Prentice, S. Marshall, S. Choglay, S. Stoffel, C. Rees, C. von Wagner

**Affiliations:** a Research Department of Behavioural Science and Health, University College London, 1-19 Torrington Place, London WC1E 7HB, United Kingdom; b St Mark’s Bowel Cancer Screening Centre, St Mark’s Hospital, Watford Road, Harrow, Middlesex HA1 3UJ, United Kingdom; c Public Health England, Skipton House, London SE1 6LH, United Kingdom; d Institute of Pharmaceutical Medicine, University of Basel, Klingelbergstrasse 61, 4056 Basel, Switzerland; e Population Health Sciences Institute, Newcastle University Centre for Cancer, Newcastle University, Newcastle Upon Tyne, United Kingdom; f Department of Gastroenterology, South Tyneside NHS Foundation Trust, South Shields, United Kingdom

**Keywords:** Flexible sigmoidoscopy screening, Colorectal cancer screening, Bowel scope screening, Reminders, Uptake

## Abstract

The clinical effectiveness of screening is highly dependent on uptake. Previous randomised controlled trials suggest that non-participant reminders, which highlight the opportunity to re-book an appointment, can improve participation. The present analysis examines the impact of implementing these reminders within the English Flexible Sigmoidoscopy (FS) Screening Programme, which offers once-only FS screening to adults aged 55–59 years.

We assessed the screening status of 26,339 individuals invited for once-only FS screening in England. A total of 10,952 (41.6%) had attended screening, and were subsequently ineligible. The remaining 15,387 had not attended screening, and were selected to receive a reminder, 1–2 years after their invitation. Descriptive statistics were used to assess the increase in uptake and the adenoma detection rate (ADR) of those who self-referred, six months after the delivery of the final reminder. Pearson’s Chi-Square was used to compare the ADR between those who attended when invited and those who self-referred.

Of the 15,387 adults eligible to receive a reminder, 13,626 (88.6%) were sent a reminder as intended (1,761 were not sent a reminder, due to endoscopy capacity). Of these, 8.0% (n = 1,086) booked and attended an appointment, which equated to a 4.1% increase in uptake from 41.6% at baseline, to 45.7% at follow-up. The ADR was significantly higher for those who self-referred, compared with those who attended when invited (13.3% and 9.5%, respectively; *X*^2^ = 16.138, *p* = 0.000059).

The implementation of non-participant reminders led to a moderate increase in uptake. Implementing non-participant reminders could help mitigate the negative effects of COVID-19 on uptake.

## Highlights

• Long-term self-referral reminders increased uptake by 4.1%.
• 13% of those who self-referred had pre-cancerous lesions detected.
• The biggest effects were seen in the most deprived areas.

## 1 Introduction

When offered as a one-off test, between the ages of 55 and 64 years, flexible sigmoidoscopy (FS) screening has been shown to reduce colorectal cancer (CRC) incidence by up to 32%, and CRC mortality by up to 50% (Elmunzer et al., 2012). As with any screening test, however, the public health benefits of once-only FS screening are highly dependent on uptake (Geurts et al., 2015). In England, where flexible sigmoidoscopy screening is offered as a one-off test to 55–59 year olds (individuals are offered an appointment at the age of 55, but can self-refer up until the age of 60), uptake is low (i.e. 43%), particularly within the most socioeconomically deprived areas (i.e. 33%), where people are most likely to benefit from having the test (McGregor et al., 2016, Aarts et al., 2010). Similar observations have been made in other countries offering and piloting FS screening. For example, in Italy, where FS screening is being piloted in several regions, uptake ranges from 29.9% in Turin, to 39.3% in Verona (Senore et al., 2013).

Questionnaires and interviews conducted with non-participants have found that ‘forgetting’ and ‘not having enough time’ are common barriers to FS screening (Hall et al., 2016, von Wagner et al., 2019). As a result, researchers have suggested that reminders, which highlight the opportunity to book a new appointment, could improve participation (Hall et al., 2016, von Wagner et al., 2019). Subsequent randomised controlled trials (RCTs) of these interventions have found them to be moderately effective, facilitating uptake in 10–20% of recipients, and adenoma detection in about ~ 8% of those who self-refer (Kerrison et al., 2018, Kerrison et al., 2017).

To date, RCTs of non-participant reminders have given little consideration towards implementation (Kerrison et al., 2018, Kerrison et al., 2017). For example, in one study conducted by Kerrison and colleagues (2016), reminders were only sent to 19% of the eligible population (Kerrison et al., 2016). For non-participant reminders to have a significant impact on uptake and clinical outcomes, they would need to be sent to a much larger proportion of the eligible population (Geurts et al., 2015). The present implementation study set out to assess the extent to which this was possible.

## 2 Patients / material and methods

Individuals who were invited for FS screening between June 2015 and November 2017 were identified on the Bowel Cancer Screening System: an electronic system that provides up-to-date information about a person’s bowel cancer screening status. The screening status of each person was assessed to determine whether they had taken part in screening and were subsequently eligible to receive a reminder (people are only eligible to have the screening test once). Individuals whose bowel cancer screening status indicated that they had not been screened, since they received their initial invitation, were eligible to receive a reminder. Reminders were then sent out to eligible adults in weekly batches of varying sizes, with fewer reminders being delivered when capacity was reduced, and more reminders being delivered when capacity was increased (e.g. due to reduced staffing).

The reminder (Appendix 1) was a one-page letter, which highlighted that the recipient was ‘overdue’ for FS screening and could self-refer by returning an ‘appointment request slip’ (Appendix 2), or calling the bowel cancer screening programme directly. A freepost return envelope was included with the letter to minimise financial and practical barriers to participation. Similarly, the telephone number provided was a freephone number; again, minimising financial barriers to uptake. Individuals who called or returned the ‘appointment request slip’ were able to indicate a preference for the gender of the practitioner performing the test, as well as the day and time of the appointment (this was elicited verbally by the administrator for individuals who called the freephone number). The reminder also included a patient information leaflet (Appendix 3), which provided further information about the procedure.

For practical reasons, a few key changes were made to the reminder process described in previous studies (Kerrison et al., 2018, Kerrison et al., 2017, Kerrison et al., 2016). First, individuals were sent a single reminder, as opposed to two reminders (i.e. one on the anniversary of their invitation and a second four weeks thereafter; this was to ensure endoscopy capacity was not overwhelmed by self-referrals). Second, participants were given six months to self-refer, rather than three (this was to ensure all self-referred appointments were included for analysis). Finally, reminders were sent 1–2 years after the initial invitation, as opposed to on the anniversary of their invite (it was not always possible to send individuals a reminder on the anniversary of their invitation, due to fluctuations in endoscopy capacity [e.g. annual leave]; this ultimately led to some individuals receiving their reminder later than others [the mean number of days between the initial invite and the reminder was 559, the range was 365–702]).

At the end of the study (September 2019), six months after the delivery of the final reminder, data on the gender and area-level deprivation of each person who received a reminder were extracted from the BCSS. Area-level deprivation was derived from the postcode of each person’s home address using the Index of Multiple Deprivation (IMD; the Governments official measure for area-level deprivation), and then categorised into quintiles based on the national distribution of neighbourhoods in England (individuals with an IMD score of 0–8 fell within the most deprived quintile, while individuals with an IMD score of 34–63 fell within the least deprived quintile) (Office of National Statistics., 2019). Data on the screening status of each person were also collected at the end of the study, along with the clinical findings of those who had attended an appointment and were screened (this was to determine uptake at ‘follow-up’).

Descriptive statistics were used to report the uptake and adenoma detection rate (ADR) of people who attended an appointment when invited and people who attended after receiving a reminder. Differences in the ADR between the two populations were assessed using Pearson’s Chi-Square (Pearson, 1900). Multivariable logistic regression was used to assess whether the ADR and uptake varied by co-variates, including: gender, area-level deprivation and response to the initial invite (individuals were categorised as ‘non-responders’ if they did not respond to the initial invite and ‘non-attenders’ if they confirmed their appointment, but subsequently did not attend).

## 3 Results

### 3.1 Baseline uptake

**Fig. 1:**
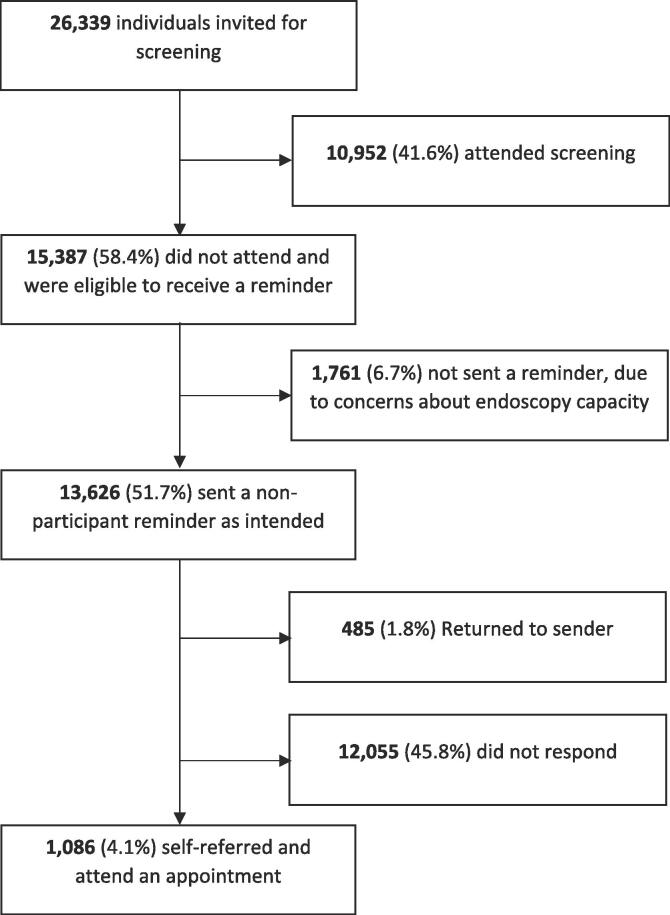
Study overview.

Between June 2015 and November 2017, 26,339 men and women were invited for FS screening. Of those, 10,952 (41.6%) had attended screening and were subsequently not eligible to receive a reminder. The remaining 15,387 adults were considered eligible. Of these, 13,626 (88.6%) were sent a reminder as intended. Only 13,141 (85.4%) of those sent the reminder, received a reminder, however, as 485 (3.2%) had moved address and the letter was ‘returned to sender’ (see Fig. 1).

### 3.2 Sample characteristics

Of the 13,626 individuals who were sent a reminder, 51.6% were men (n = 7033) and 48.4% were women (n = 6593). There was a larger number of non-responders (n = 13,018, 95.5%) than non-attenders (n = 608, 4.5%). The mean IMD score of individuals was 20.8, which is similar to the national average (21.7) (Office of National Statistics, 2019) (Table 1).

**Table 1:** Sample characteristics.

		N	(%)
**Gender**			
	Male	7033	(51.6%)
	Female	6593	(48.4%)
**Area-level deprivation** (IMD Score)			
	IMD quintile 1 (0–8, least deprived)	1558	(11.4%)
	IMD quintile 2 (9–13)	2307	(16.9%)
	IMD quintile 3 (14–20)	3730	(27.4%)
	IMD quintile 4 (21–33)	3695	(29.1%)
	IMD quintile 5 (34–63, most deprived)	1867	(13.7%)
	Missing	199	(1.5%)
**Baseline screening status**			
	Non-responder	13,018	(95.5%)
	Non-attender	608	(4.5%)

IMD = Index of Multiple Deprivation.

### 3.3 Follow-up uptake

Of those who were sent a reminder (n = 13,626), 1,086 (8.0%) booked and attended an appointment. This increased the overall uptake of the sample from 41.6% (10,952 / 26,339), to 45.7% (12,038 / 26,339), which equated to an absolute increase of 4.1%. The reminder was not significantly more effective at facilitating uptake for people living in the least socioeconomically deprived quintile of areas, compared with people living in the most deprived quintiles (uptake was 8.3% and 7.4%, respectively; aOR: 0.87, 95%CIs: 0.68, 1.88, *p* = 0.262); however, the results were different when area-level deprivation was entered into the model as a continuous variable, with increased area-level deprivation being associated with reduced odds for attending screening – see Appendix 4). The reminder was also more effective for previous non-attenders than previous non-responders (uptake was 10.9% and 7.9%, respectively; aOR: 1.44, 95%CIs: 1.11, 1.88, *p =* 0.007), but there were no statistically significant differences in uptake between women and men (uptake was 7.9% and 8.0%, respectively; aOR: 1.02, 95%CIs: 0.90, 1.15, *p* = 0.701; Table 2).

**Table 2:** Variation in uptake and adenoma detection rate by sample characteristics (univariable and multivariable logistic regression outcomes).

	Uptake of CRC screening							Adenoma detection						
	N	(%)	Unadjusted model			Adjusted model		N	(%)	Unadjusted model			Adjusted model	
Variable			OR	95% CI		OR	95% CI			OR	95% CI		OR	95% CI
**Gender**														
Female	521	(7.9%)	Ref.			Ref.		48	(9.2%)	Ref.			Ref.	
Male	565	(8.0%)	1.02	0.90–1.15		1.03	0.90–1.16	96	(17.0%)	2.02	**1.39**–2.92**		2.08	**1.43**–3.02**
**Area-level deprivation**														
IMD quintile 1 (least deprived)	130	(8.3%)	Ref.			Ref.		11	(8.5%)	Ref.			Ref.	
IMD quintile 2	210	(9.1%)	1.10	0.88–1.38		1.10	0.87–1.38	29	(13.8%)	1.73	0.83–3.60		1.84	0.88–3.84
IMD quintile 3 (least deprived)	307	(8.2%)	0.99	0.80–1.22		0.98	0.79–1.22	35	(11.4%)	1.39	0.68–2.83		1.48	0.73–3.03
IMD quintile 4	290	(7.3%)	0.87	0.70–1.08		0.86	0.70–1.07	43	(14.8%)	1.88	0.94–3.78		1.93	0.96–3.88
IMD quintile 5 (most deprived)	138	(7.4%)	0.88	0.68–1.13		0.87	0.68–1.88	25	(18.1%)	2.39	1.13–5.09*		2.49	**1.16**–5.34*
**Baseline screening status**														
Non-responder	1,020	(7.9%)	Ref.			Ref.		134	(13.1%)	Ref.			Ref.	
Non-attender	66	(10.9%)	1.43	1.10–1.86**		1.44	1.11–1.88**	10	(15.2%)	1.18	0.59–2.37		1.17	0.57–2.40
***N =*** 13,427														

IMD = Index of Multiple Deprivation, OR = Odds ratio, aOR = adjusted odds ratio, 95% CI = 95% confidence interval, CRC = Colorectal cancer screening.

*p < 0.05; **p < 0.01.

### 3.4 Clinical outcomes

The ADR for people who attended when invited was 9.5% (1,036 / 10,952), while the ADR for people who attended after receiving a reminder was 13.3% (144 / 1,086), which was significantly higher (*X*^2^ = 16.138, *p* = 0.000059). The proportion with high or intermediate risk adenomas, specifically, was similar between the two groups (1.8% [n = 19] of those who attended after receiving a reminder had intermediate or high risk adenomas, compared with 2.1% [n = 233] of those who attended when invited; *X*2 = 0.689, *P* = 0.406664). The proportion with low risk adenomas, specifically, however, was significantly higher among those who self-referred (11.5% [n = 125] of those who attended after receiving a reminder had low risk adenomas, compared with 7.3% [n = 803] who attended when invited; *X2* = 24.2427, *P <* 0.001).

Among those who received a reminder, the ADR was higher among men than women (17.0% vs 9.2%, respectively; aOR: 2.08, 95%CIs: 1.43, 3.02, *p* < 0.001) and people living within the most deprived quintile of areas, compared with the least deprived quintile of areas (18.1% vs. 8.5%, respectively; aOR: 2.49, 95%CIs: 1.16, 5.34, *p* = 0.019; the results were the same when area-level deprivation was entered into the model as a continuous variable – see Appendix 4).

The ADR observed for men and women who attended when invited (i.e. 9.2% and 17.0%, respectively) is similar to the ADR recently reported for men and women (i.e. 11.5% and 6.6% for men and women, respectively) in an analysis of the national screening programme (Bevan et al., 2019). There were no statistically significant differences in the ADR between non-responders and non-attenders (13.1% vs. 15.2%, respectively; aOR: 1.17, 95%CIs: 0.57, 2.40, *p* = 0.666).

## 4 Discussion

### 4.1 Summary of findings

This study assessed the clinical effectiveness of implementing non-participant reminders within the English Bowel Cancer Screening Programme. It found that, despite changes to the reminder, non-participant reminders facilitated uptake in 8% of recipients, increasing uptake from 41.6% at baseline, to 45.7% at follow-up (an absolute increase in uptake of 4.1%, and a relative increase in uptake of 11%). It also found that uptake was higher among previous non-attenders than previous non-responders, as well as individuals living in less deprived areas. While this would appear to exacerbate existing inequalities in uptake, it is important to remember that a greater proportion of individuals living within the most deprived quintile of areas are non-participants (approximately 67% of individuals living within the most deprived quintile of areas are non-participants, whereas only 47% of individuals living within the least deprived quintiles of areas are non-participants (McGregor et al., 2016). As such, the greatest gains in participation would most likely be observed in the most deprived quintile of areas, as opposed to the least deprived quintile of areas, were these interventions implemented (with uptake increasing by 5.0% in the most deprived quintile, and only 3.9% in the least deprived quintile; estimates calculated by multiplying the proportion of non-participants in the most and least deprived quintiles of areas [i.e. 0.67 and 0.37, respectively], by the proportion who take up screening after receiving a reminder within those quintiles [i.e. 0.074 and 0.083, respectively]).

Importantly, this study also found that the ADR was higher for adults who self-referred, compared with individuals who attended when invited, suggesting that the interventions are facilitating uptake in individuals who are at risk of developing bowel cancer in the future.

### 4.2 Comparisons with the previous literature

The results of our study compare favourably with many large trials that have led to changes in health policy by achieving more modest increases in uptake. For example, the ASCEND trial, which examined the effectiveness of interventions to promote the uptake of faecal occult blood test (FOBT) screening, led to the inclusion of a GP endorsement on the invitation letter, on the basis that it led to an absolute increase in uptake of 0.7% (Wardle et al., 2016, Raine et al., 2016). Similarly, an enhanced reminder letter for FOBT screening, which added the heading ‘A reminder to you’ and a short paragraph restating the test offer of screening in simple language, was implemented on the basis that it too led to an absolute increase of 0.7% (Raine et al., 2016).

The finding that non-participant reminders were more effective for non-attenders than non-responders is consistent with previous studies investigating non-participant reminders (Kerrison et al., 2018, Kerrison et al., 2017). We have previously hypothesised that the reasons why non-participant reminders are more effective for non-attenders is that they: 1) perceive fewer barriers to screening, making it easier for them to book and attend an appointment and, 2) have already formed the intention to go for screening, but have previously cancelled due to circumstantial events (e.g. unwell on the day of the appointment) (von Wagner et al., 2019). These hypotheses are supported by two recent studies exploring predictors of intention-translation (von Wagner et al., 2019) and reasons for non-attendance (Hall et al., 2016): the former found that non-attenders prospectively report fewer barriers to FS than their non-responding counterparts, while the latter confirmed that unexpected events, including illness, were frequently cited reasons for non-attendance. Additional interventions, which help form intentions, may, consequently, be required to facilitate uptake among consistent non-responders.

The finding that individuals from less socioeconomically deprived areas are more likely to book and attend an appointment is also consistent with previous research (McGregor et al., 2016). As with the differences between non-responders and non-attenders, previous studies have shown that individuals living in the most socioeconomically deprived areas report more barriers to participation than individuals living in the least deprived areas, making it more difficult for them to attend (Whitaker et al., 2011).

To our knowledge, the finding that people who self-refer for FS screening are more likely to have adenomas detected has not previously been reported. There are two possible explanations as to why these individuals are more likely to have adenomas. The first is that individuals who were sent a reminder and self-referred for screening are slightly older (the reminder was sent 1–2 years after the initial invitation) (Brenner et al., 2015). This explanation seems unlikely to account for all of the variance, however, given that previous research suggests the ADR increases from 9% between the ages of 50 and 59 years, to 11% between the ages of 60 and 69 (Atkin et al., 1993). The second is that this group may have poorer health behaviours and are therefore more at risk of developing adenomas than those who attend when invited (smoking, alcohol consumption, and diets high in red and processed meat and low in fruits and vegetables are all associated with an increased risk of adenoma development) (Larsen et al., 2006). The finding that it is low risk adenomas, specifically, which individuals who receive a reminder are more likely to have detected, suggests that these are new adenomas, which have developed during the interval.

Finally, it is important to note that we observed a slightly lower self-referral rate than seen in previous studies (8% vs. 10–20%), and not everyone who was eligible to receive a reminder was sent one (Kerrison et al., 2018, Kerrison et al., 2017). The most likely explanation as to why we observed a slightly lower self-referral rate, is that we only used one reminder, as opposed to two (a previous feasibility study indicated that the self-referral rate was 10% prior to the delivery of the first reminder and increased to 15% after the delivery of the second) (Kerrison et al., 2016).

### 4.3 Strengths and limitations

This study had several strengths. First, it used objective data to assess attendance at FS screening. Second, it contained a large sample size, allowing more accurate estimates to be produced. Finally, it allowed the real-world impact of the interventions to be established, by including the invited population, and not just those who received a reminder, as was the case with previous studies (Kerrison et al., 2018, Kerrison et al., 2017, Kerrison et al., 2016).

The present study also had several limitations. First, an area-level measure for socioeconomic deprivation was employed, as opposed to an individual-level measure of socioeconomic deprivation. Second, demographic data were only extracted for those who received a reminder, and not the entire population (permission to extract demographic data on those who had attended was not obtained). Finally, the study was restricted to data available on the bowel cancer screening system. As such, it was not possible to include known psychological variables as co-variates in the regression models, nor to deliver the reminder via any format other than post (patient telephone numbers are not available for non-participants), which may have be a less effective method than a telephone or text message reminder.

### 4.4 Implications for policy

At the time of writing, there is a novel coronavirus pandemic (i.e. SARS CoV-2), which has led to the temporary suspension of screening services in England (March 2020). One of the concerns expressed by expert groups is that, when endoscopy services resume, patients will be reluctant to attend, due to fears of contracting or spreading the virus (this is already being observed for colonoscopy appointments offered to people who had an abnormal faecal immunochemical test result). The implementation of non-participant reminders may help mitigate a potential rise in CRC cases and deaths (expected as a result the suspension of screening services and reduced primary care referrals) by increasing FS screening uptake. It is our belief, therefore, that they should be implemented as soon as screening services resume.

## 5 Conclusions

The implementation of non-participant reminders led to a moderate increase in uptake. Importantly, the implementation of non-participant reminders led to uptake in a high proportion of people with adenomas. Implementing non-participant reminders nationally, once screening services resume, could help mitigate the negative effects of COVID-19 on uptake.

## 6 Ethics

Ethical approval was not required for this study, as it was considered ‘service evaluation', as opposed to ‘research’, by the NHS Health Research Authority.

## Funding

This study was funded by a commissioning for quality and innovation grant from NHS England (Ref: 17/18/001/NWL. Robert S Kerrison is supported by a Cancer Research UK Population Research Fellowship [C68512/A28209].

## CRediT authorship contribution statement

**R.S. Kerrison:** Conceptualization, Formal analysis, Investigation, Supervision, Visualization, Writing - original draft, Writing - review & editing. **A. Prentice:** Conceptualization, Data curation, Investigation, Project administration, Writing - original draft. **S. Marshall:** Conceptualization, Data curation, Investigation, Project administration, Writing - original draft. **S. Choglay:** Data curation, Investigation, Project administration, Writing - original draft. **S. Stoffel:** Formal analysis, Investigation, Validation, Visualization, Writing - original draft. **C. Rees:** Investigation, Validation, Writing - original draft. **C. von Wagner:** Conceptualization, Formal analysis, Investigation, Supervision, Writing - original draft.

## Declaration of Competing Interest

The authors declare that they have no known competing financial interests or personal relationships that could have appeared to influence the work reported in this paper.
