# Supplementary material for: Implementation of long-term non-participant reminders for flexible sigmoidoscopy screening
Source: Prev Med Rep. 2021 Jan 4;21:101308. doi: 10.1016/j.pmedr.2020.101308 (PMC7815459; doi:10.1016/j.pmedr.2020.101308)
Supplement: Supplementary data 2 [file mmc2.docx]

**IMPORTANT**: Please check your details and return in the freepost envelope provided

**Name:** «First_Name» «Surname»

**NHS Number:** «NHS_Number»

**Post Code:** «Postcode»

**Please fill in your details** (either your home telephone number or your mobile number is required; this is so we can contact you to confirm your appointment)**:**

Home telephone number:

Mobile telephone number:

Please **tick** this box if you would like to have a bowel scope screening appointment:

- I’d like to arrange a bowel scope screening appointment at St Mark’s Hospital in Harrow.

Please **tick** your preference:

- I would prefer to have a **Male** practitioner to perform my test.
- I would prefer to have a **Female** practitioner to perform my test.
- I have no preference for either male or female practitioner.

Please **tick** as appropriate from the available time slots: My preferred appointment time(s) would be:

|  | **Morning**  **8:30-11:00** | **Afternoon**  **13:00-15:30** | **Evening**  **16:45-19:00** |
| --- | --- | --- | --- |
| **Monday** |  |  |  |
| **Tuesday** |  |  |  |
| **Wednesday** |  |  |  |
| **Thursday** |  |  |  |
| **Friday** |  |  |  |
