# Supplementary material for: Implementation of long-term non-participant reminders for flexible sigmoidoscopy screening
Source: Prev Med Rep. 2021 Jan 4;21:101308. doi: 10.1016/j.pmedr.2020.101308 (PMC7815459; doi:10.1016/j.pmedr.2020.101308)

ST MARK'S HOSPITAL

NHS

One less thing to worry about

A simple screening test could prevent you from getting bowel cancer...

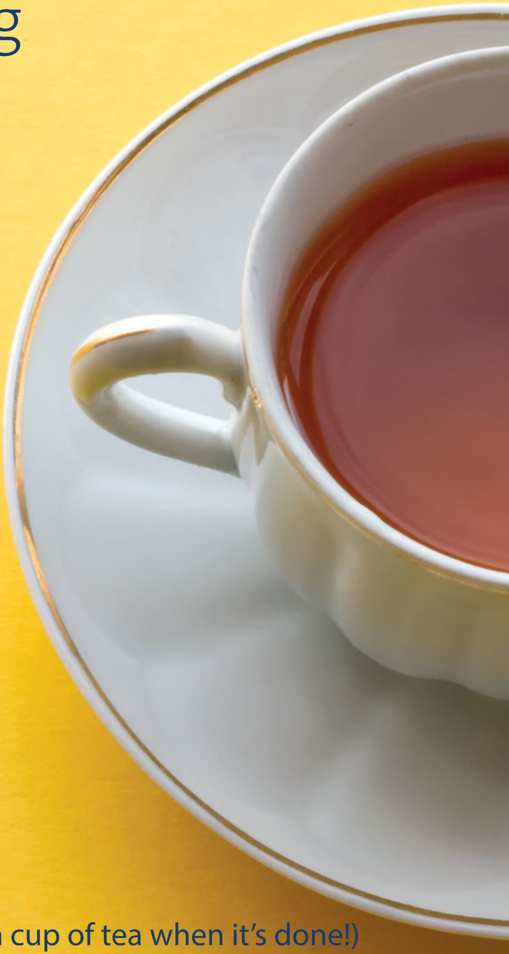

...And the NHS is inviting everyone aged 55 to 59 in your area

(It's quick and easy and we'll give you a cup of tea when it's done!)

The bowel scope screening test is quick, easy and you only do it once, but it could be a lifesaver. So book your appointment now.

Why this test is for you

Bowel cancer is Britain's third most common cancer – and our second biggest cancer killer.

We're contacting you because after the age of 55, your risk of bowel cancer increases. But screening can cut your chance of getting the disease, dramatically.

Everyone aged 55 to 59 is receiving this invitation. You don't need to have any symptoms or family history of bowel cancer to take part – most people who have the test don't.

"I'd never thought about bowel cancer – no one in my family has had it and I'm pretty fit and well. But I took the test, to get it over with really. It was very straightforward and it's comforting to know that everything's ok."

Recent patient, 56 yrs, Kensal Green in Brent

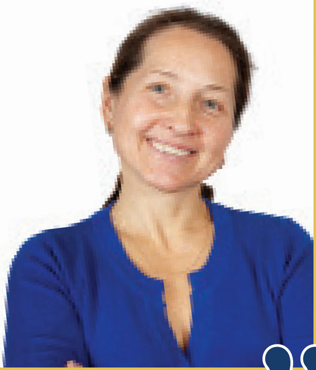

What happens next?

Call us on 0800 707 6060 and book your appointment at a time convenient to you. Your appointment will be at St Mark's, Northwick Park Hospital - find out more at www.stmarkshospital.nhs.uk

Bowel scope screening involves a simple procedure called Flexible Sigmoidoscopy. It uses a camera on a flexible tube to examine your lower bowel for small growths called polyps. Removing any polyps can dramatically reduce your chance of getting bowel cancer.

Before the test you'll need an enema to make sure your bowels are empty. This is something you can easily do at home – you'll receive a pack in the post. If you prefer though, you can bring your enema to the hospital, where we can help you.

Watch Dr XXXXX explain more about bowel scope screening and hear what people who've had the test are saying about it at: www.stmarkshospital.nhs/videos

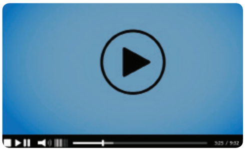

What you need to know

- ✔ This test is for **everyone** aged 55 to 59
- ✔ It's **not just** for people with symptoms or a family history
- ✔ It's a **simple, 20-minute** procedure called Flexible Sigmoidoscopy
- ✔ It provides **peace of mind**, and could be life-saving
- ✔ It can **dramatically reduce** your risk of getting Bowel Cancer

Call **0800 707 6060** if you have any questions and to make your appointment today Our phone lines are open (8am to 5pm Monday to Friday, 8am to 4pm on Saturdays and 9am to 4pm on Sundays).

St Mark's, at Northwick Park Hospital

St Mark's is easy to reach by tube, bus and car. Northwick Park tube station (Metropolitan line) is only five minutes walk away. See www. tfl.gov.uk for more information on reaching the hospital.

Map and location

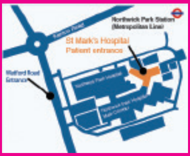

St Mark's Hospital
Northwick Park,
Watford Road
Harrow, Middlesex
HA1 3UJ

Contact us

If you have any questions about bowel scope screening, please give our team a call on 0800 707 6060. We are open 8am to 5pm Monday to Friday, 8am to 4pm on Saturdays and 9am to 4pm on Sundays.

"If you're aged 55 to 59 this is one thing you shouldn't ignore. I urge you to take this quick and potentially life-saving test. It's a one off chance to significantly reduce your risk of getting bowel cancer."

Dr Stephen Mort, cancer lead for Hillingdon Church Road Surgery.

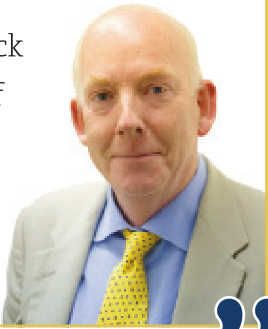

Supplement: Supplementary data 3 [file mmc3.pdf]
