# Supplementary material for: Implementation of long-term non-participant reminders for flexible sigmoidoscopy screening
Source: Prev Med Rep. 2021 Jan 4;21:101308. doi: 10.1016/j.pmedr.2020.101308 (PMC7815459; doi:10.1016/j.pmedr.2020.101308)
Supplement: Supplementary data 4 [file mmc4.docx]

| **Appendix 4.** Variation in uptake and adenoma detection rate by sample characteristics (univariable and multivariable logistic regression outcomes) | | | | | | | | | | |
| --- | --- | --- | --- | --- | --- | --- | --- | --- | --- | --- |
|  | **Uptake**  ***n (%)*** | **OR**  **(95% CIs)** | ***p*** | **aOR**  **(95% CIs)** | ***p*** | **ADR**  ***n (%)*** | **OR**  **(95% CIs)** | ***p*** | **aOR**  **(95% CIs)** | ***p*** |
| **Sex** | | | | | | | | | | |
| Women | 521  (7.9) | 1.00 | - | 1.00 | - | 48  (9.2) | 1.00 | - | 1.00 | - |
| Men | 565 (8.0) | 1.02  (0.90, 1.15) | 0.777 | 1.02  (0.90, 1.16) | 0.707 | 96 (17.0) | 2.02  (1.39, 2.92) | **<0.001** | 2.07  (1.42, 3.00) | **<0.001** |
| **Area-level deprivation** | | | | | | | | | | |
| IMD score (0-63) | - | 0.99  (0.99, 1.00) | **0.015** | 0.99  (0.99, 1.00) | **0.011** | 27  (10.5) | 1.02  (1.00, 1.03) | **0.017** | 1.02  (1.00, 1.03) | **0.018** |
| **Baseline screening status** | | | | | | | | | | |
| Non-responder | 1020  (7.8) | 1.00 | - | 1.00 | - | 134  (13.1) | 1.00 | - | 1.00 | - |
| Non-attender | 66  (10.9) | 1.43  (1.10, 1.86) | **<0.001** | 1.45  (1.11, 1.89) | **0.006** | 10  (15.2) | 1.18  (0.59, 2.37) | 0.640 | 1.17  (0.57, 2.38) | 0.669 |
| IMD = Index of Multiple Deprivation, OR = Odds Ratio, aOR = Adjusted Odds Ration, 95%CIs = 95% Confidence Intervals, ADR = Adenoma Detection Rate | | | | | | | | | | |
